# Towards sustainable charcoal production: Designing an economical brick kiln with enhanced emission control technology

**DOI:** 10.1016/j.heliyon.2024.e27797

**Published:** 2024-03-08

**Authors:** Zelalem Getahun, Mikiyas Abewaa, Ashagrie Mengistu, Eba Adino, Kumera Kontu, Kenatu Angassa, Amare Tiruneh, Jemal Abdu

**Affiliations:** a Department of Chemical Engineering, College of Engineering and Technology, Wachemo University, Hossana, Ethiopia; b The Federal Democratic Republic of Ethiopia, Manufacturing Industry Institute, Addis Ababa, Ethiopia; c Department of Chemical Engineering, School of Mechanical, Chemical and Materials Engineering, Adama Science and Technology University, Adama, Ethiopia; d Department of Mechanical Engineering, College of Engineering and Technology, Dembidolo University, Dembidolo, Ethiopia; e Department of Environmental Engineering, College of Engineering, Addis Ababa Science and Technology University, Addis Ababa, Ethiopia

**Keywords:** Bricks, Charcoal, Carbonization, Scrubber, Emission gas control

## Abstract

In this research, a brick kiln integrated with pollutant emission control technology was designed and applied in order to produce charcoal from Eucalyptus Globules wood. The batch operation carbonization of wood biomass was undertaken in a 1.25 $m^{3}$ volume brick kiln. A wet-packed scrubber was designed and constructed by filling gravels in a depth of 40 cm with aggregate sizes of 48–60 mm, 27–33 mm and 16–20 mm from the bottom to the top respectively aiming to treat emission from the charcoal-producing unit. The characteristics of the charcoal produced were determined to be composed of 9% moisture content, 1.5% ash content, 38% charcoal yield and a heating value of 27.53 MJ/kg. On the other hand, the wet scrubber integrated into a brick kiln was found to remove Hydrocarbons, ${CO}_{2}$ and CO by 97.8%, 98.5% and 99% respectively, which makes it efficient and practical way of controlling the gasses released during producing of charcoal.

## 1 Introduction

Charcoal is a widely used source of fuel that is obtained from carbonization of organic materials such as wood and other biomass. These organic materials are chosen based on their carbon content, availability, and suitability for the production of high-quality charcoal [1]. Wood, being the most common raw material, offers a sustainable source for charcoal production. In tropical regions, coconut shells are abundant and are known for their high carbon content, making them a valuable resource for charcoal manufacturing [2]. Other organic matter such as peat that found in wetlands is also utilized in some regions for charcoal production [3].

The use of charcoal spans across various industries and applications. One of the primary uses of charcoal is in cooking and heating. It is favored for grilling and barbecuing due to its high heat output, long burn time, and ability to impart a smoky flavor to the food. In a study [4], it was found that charcoal briquettes made from agricultural waste showed promising results as a clean and efficient cooking fuel, reducing emissions and improving indoor air quality. Charcoal is also widely used in metallurgical processes, such as iron and steel production, due to its high carbon content and low impurities [5,6]. Moreover, charcoal finds applications in water and air purification systems. Research by Ref. [7] demonstrated that activated charcoal effectively removes pollutants and contaminants from water sources, making it a valuable tool for water treatment.

Worldwide charcoal consumption has been a significant energy and cooking fuel source, particularly in regions where access to modern energy services is limited. As per the latest report provided by report from the Food and Agriculture Organization (FAO), global charcoal production reached approximately 53 million metric tons in 2020, with the majority of consumption occurring in Africa. Africa, in particular, has a high reliance on charcoal for cooking, with an estimated 80% of urban households and 90% of rural households using charcoal as their primary cooking fuel [8]. This heavy reliance on charcoal stems from factors such as limited access to electricity and clean cooking alternatives, as well as affordability and cultural preferences for charcoal cooking methods. In Africa, Ethiopia is one of the largest consumers of charcoal on the continent. The consumption of charcoal in Ethiopia has witnessed a significant increase in recent years due to rapid urbanization and population growth. The World Bank estimates that around 60% of urban households in Ethiopia depend on charcoal as their main cooking fuel [9,10]. The demand for charcoal in urban areas has led to deforestation and environmental degradation, as unsustainable production practices are often employed to meet the growing demand. The Ethiopian government has recognized the need to address these challenges and has implemented various initiatives to promote sustainable charcoal production and alternative cooking solutions, such as improved cook stoves and electrification programs.

Charcoal production methods vary significantly, ranging from traditional and rudimentary techniques to more advanced and efficient processes. The efficiency of these methods has important implications for the quality and quantity of charcoal produced in addition to the environmental impact of production. Traditional methods, such as earth mound kilns and pit kilns, are commonly used in many regions but are known for their low efficiency and high emissions of pollutants. In contrast, advanced production methods, including retort kilns and pyrolysis technologies, have emerged as more efficient alternatives that offer higher yields of charcoal and reduced environmental impact [[11], [12], [13]]. A study by Ref. [14] compared the efficiency of traditional pit kilns with advanced retort kilns for charcoal production. The results showed that retort kilns achieved significantly higher charcoal yields and reduced emissions compared to pit kilns. Retort kilns operate in a closed system, which allows for the recovery of by-products and minimizes the release of volatile compounds and pollutants into the atmosphere. Additionally, the study found that retort kilns produced charcoal with higher carbon content and lower impurities, resulting in improved quality and energy density. Another technology gaining attention is pyrolysis, which involves the heat-induced breakdown of biomass without the presence of oxygen. Pyrolysis processes, such as fast pyrolysis and torrefaction, have shown promise in enhancing the efficiency of charcoal production. Research by Ref. [15] demonstrated that fast pyrolysis of biomass can yield higher charcoal production rates and reduce emissions compared to traditional methods. Furthermore, torrefaction, a mild pyrolysis process, has been explored as a means to improve the energy density and stability of charcoal.

The environmental implications of different charcoal production methods are significant. Traditional methods often involve the unsustainable harvesting of wood resources, leading to deforestation and habitat degradation. Moreover, the incomplete combustion in traditional kilns releases substantial amounts of greenhouse gases, particulate matter, and volatile organic compounds which contribute to air pollution and climate change [16,17] Advanced production methods, such as retort kilns and pyrolysis technologies, offer more efficient use of feedstock and reduce emissions due to their closed systems and optimized combustion processes [13,18]. These advancements contribute to mitigating effects on the environment, like deforestation, carbon emissions, and air pollution related to charcoal production.

The need for cheaper and environmentally sustainable charcoal production methods has become increasingly evident in recent years, driven by concerns over deforestation, carbon emissions, and the negative impacts on ecosystems and local communities [[19], [20], [21]]. Traditional charcoal production methods often involve the unsustainable harvesting of wood resources, leading to deforestation and habitat degradation. Furthermore, traditional kilns used in charcoal production typically operate with low efficiency and emit significant amounts of greenhouse gases, particulate matter, and volatile organic compounds into the atmosphere [22,23]. There is an increasing need to address these issues by providing a cheaper and environmentally sustainable charcoal making techniques.

One approach to achieving cheaper and sustainable charcoal production is through the adoption of advanced technologies such as retort kilns and pyrolysis processes. Research by Ref. [14] has shown that advanced retort kilns offer higher charcoal yields, reduced emissions, and improved quality compared to traditional kilns. These closed-system kilns recover by-products and minimize the release of pollutants, making them more environmentally friendly. Similarly, pyrolysis processes, such as fast pyrolysis and torrefaction, have demonstrated potential for more efficient and sustainable charcoal production [15]. Highlighted the benefits of fast pyrolysis in terms of higher charcoal production rates and reduced emissions. However, most of the poor households don't afford the relatively expensive retort kiln and pyrolysis process and hence, the need to develop and design low cost and sustainable charcoal making technology is needed. To this end, this piece of work focused on design and development of affordable brick kiln integrated with emission control technology.

## 2 Materials and methods

### 2.1 Study area

This research was carried out in Ethiopia, Oromia Region, specifically, Balemi Kebele, situated 220 km west of Addis Ababa. Calliya wereda, Jimma Rare, Tokke Kutaye and Ambo Wereda, and Gindeberet are the neighbouring Weredas for Balemi Kebele by south, west, east and by north respectively. Since the altitude of Balemi is around 2000 m above sea level, it has around six month's rainy season like other highland parts of Ethiopia. Agriculture serves as the primary means of generating income.as it covers around 90%. In this particular administrative division, the cultivation of various bowls of cereal is predominantly practiced. However, for some part of the society charcoal is other source of income. It is being produced from native trees. Moreover, there is high surface and underground water; hence there is high tendency of eucalyptus tree in this location. Therefore, this location was selected for its appropriate access for the project.

### 2.2 Materials

**Fig. 1:**
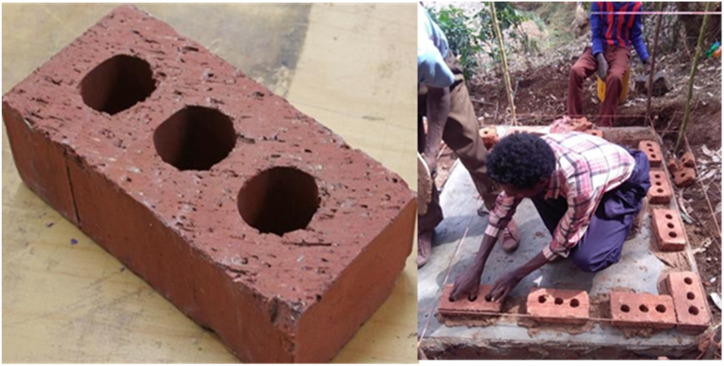
Brick utilized for construction of kiln.

Raw materials like Bricks, gravels, wood and auxiliary materials like cement, metal sheet and water were used in this research. Brick is chosen as the preferred material for constructing the kiln used in charcoal production due to its durability and ability to withstand high temperatures [24] as shown in Fig. 1. For this research purpose, [25 × 12 × 6 cm] burned bricks were utilized, they were acquired from the Ethio-Bricks industry.

**Fig. 2:**
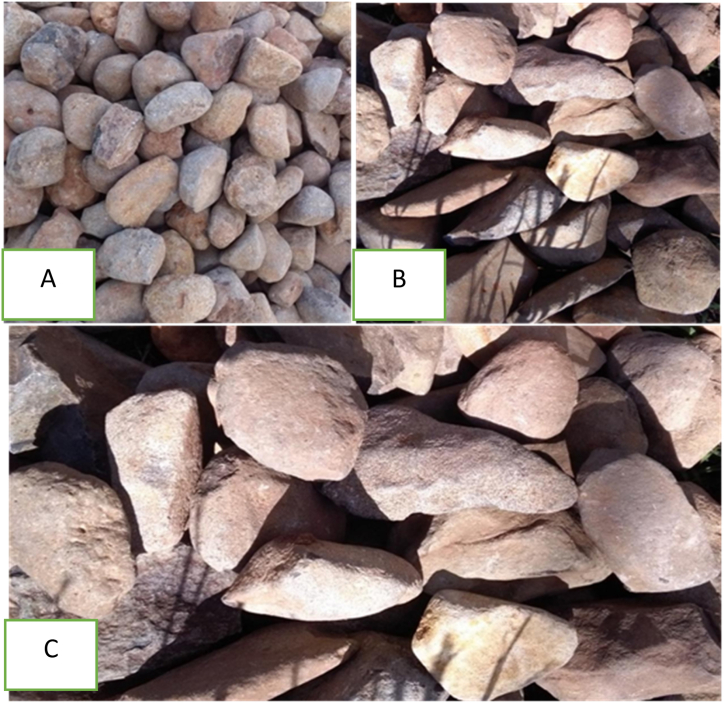
Gravel type with sizes of 16–20 mm (A), 27–33 mm (B) and 48–60 mm (C).

The pebble river gravel utilized in this particular research possesses an aggregate size of 16 mm–20 mm, 27 mm–33 mm and 48 mm–60 mm where the configuration of the top, middle, and bottom is set in place. Gravel was selected because of its washable property to be used for many cycles and its inert nature. Moreover, gravel was employed as a supportive medium in order to enhance the interaction between smoke and water/solutions, thereby maximizing efficiency as shown in Fig. 2 (A, B and C) [25].

In this work, Eucalyptus globules wood has been chosen. This species is widely distributed throughout Ethiopia's highlands, with estimates of its covered land reaching 506,000 ha prior to 15 years [26]. After four months of drying, the amount of water content of the size reduced wood decreased to 29.5%. Additionally, various auxiliary materials such as cement for the purpose of binding the bricks, metal sheets for constructing the chimneys, a water tanker, a wet packed scrubber tower, and openings of the kiln were utilized in this research. Besides this, water is used to absorb pollutants and to wash away trapped particulate matter and other hydrocarbons from the gravel. Finally, equipment like a gas detector for detecting gases such as carbon monoxide, carbon dioxide, nitrogen oxides, and sulfur oxides, and a gas analyzer for analysing carbon monoxide, and hydrocarbons were used.

### 2.3 Kiln design and construction

**Fig. 3:**
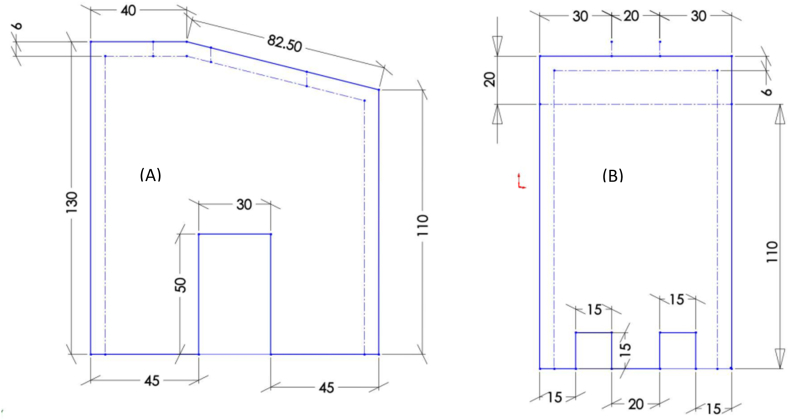
Kiln design (A) front and (B) left side view.

**Fig. 4:**
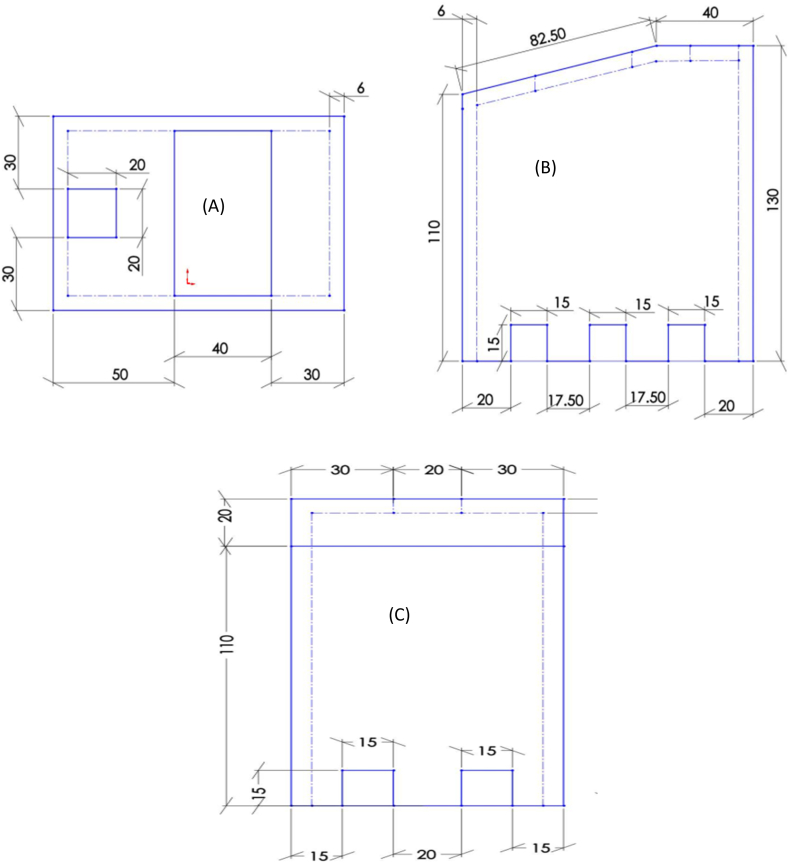
Kiln design views; (A), Top view (B) Rear view and (C) Right side view.

Various shapes of brick kilns are employed for the process of producing charcoal in batches and can be fabricated in diverse forms, such as the beehive kiln in Brazil [27], the half orange kiln in Argentina [28], the rectangular kiln known as Adam retort [29], the Missouri kiln and other variations [30]. Besides this, kilns are constructed with varying measurements and configurations according to the desired capacities. For example, small-sized kilns are usually designed to produce 4–5 bags while, larger one kilns' design is based on 80–120 bags production capacity. Different scholars have affirmed that the thickness of the kiln walls ranges from 30 to 40 cm for smaller kilns and from 42 to 48 cm for larger ones, with the purpose of safeguarding the wood carbonization process from excessive heat dissipation [31]. In this particular investigation, the criteria employed to determine the kiln dimensions were twofold. Firstly, the volume of wood to be tested in a one batch was taken into account, while reserving 20% of the kiln's total volume for the empty space above the wood. Secondly, the decision to opt for a rectangular kiln was motivated by its inherent simplicity. Furthermore, the Adam resort kiln having a rectangular design was selected owing to its high efficiency. Therefore, kiln dimensions were selected to be 156 cm × 116 cm × 146 cm (while internal dimensions 120 × 80 × 130 cm) as shown in Fig. 3 A and B. The kiln was constructed using the subsequent procedures: Initially, levelling off the ground was done. Then, the kiln was built with air inlets at the base while a hole/chimney which controls the outflow of gases was placed at top of one end side. Additionally, the kiln was built as a pilot test with an internal dimension of 0.8 m × 1.2 m x 1.3 m (W × L × H) which gave a 1.25 $m^{3}$ volume. The construction of the brick kiln took two days utilizing 500 pieces of bricks. The kiln was allowed to have one opening on top for wood charging and closed after carbonization of wood was initiated. Additionally, on the kiln's front wall, there is a small metal door for the discharge of charcoal as shown in Fig. 4 A, B and C.

### 2.4 Wet packed scrubber design

Wet packed scrubbers are air pollution control devices that use liquid to absorb gaseous pollutants and remove solid particles from gas streams. They are preferred due to their simplicity in design, low energy consumption, and ability to handle large volumes of gases. In this process, Gaseous pollutants such as CO and CO_2_ from combustion are absorbed by particulate matter stuck to gravel and water droplets, whereas gravel is rinsed by water flowing downstream. Subsequently, as emissions reach the base of the tower, a contaminated airstream rises across wetted packing (gravel). Then, the downward flowing liquid evenly spreads along the packing of the column whereby the total area of contact between the gas and liquid is increased. Most wet packed scrubbers are based on cylindrical design. However, a rectangular design is selected in this study owing to its ease of construction.

The device's design was based on various assumptions and data from a kiln, revealing that 133.2 kg from 223 kg of wood was released into the environment during carbonization. It took 16 h to finish this process. As a result, the determined mass flow rate (mf) was 133.2 kg/16 h, or 8.325 kg/h. The other factor that goes into determining the tower's measurements is the effective density of exhaust gas released from the kiln during the carbonization stage. As a result, during the steady combustion phase, tiny particle agglomerates are generated with an effective density of around 1 g/cm^3^. Equations (1)–(4) are used during designing the wet scrubber design.

From ideal gas law, PV = nRT.

Can be solved for the gas volume to get:

(1) $$V=\frac{nRT}{P}$$

Density of the gas is determined by:

(2) ρ=m/V

when equation (1) is substituted into equation (2), gas density is redefined as:

(3) $$\rho =\frac{mP}{nRT}$$

Consequently, density multiplied by volume flow rate equals mass flow rate:

(4) $$\text{mf}=\rho \times V_{f}$$

**Fig. 5:**
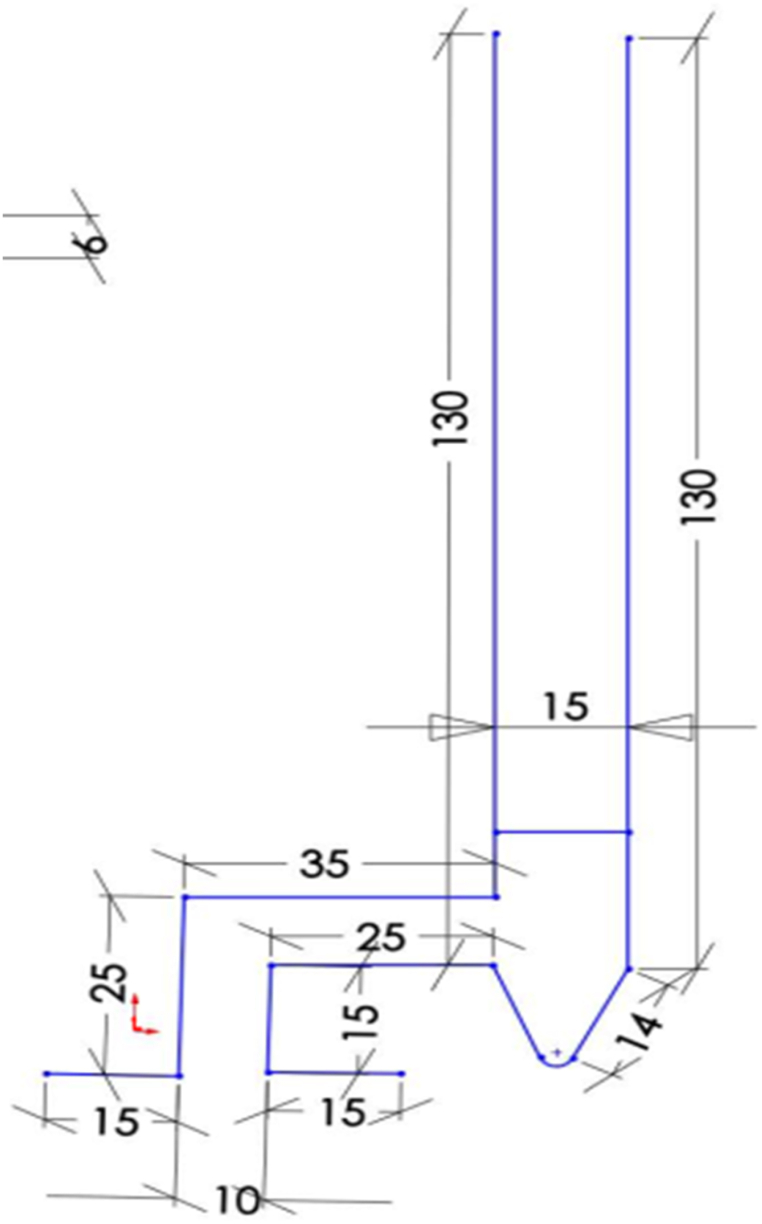
Wet packed scrubber designed for controlling pollutants emitted from charcoal producing brick kiln.

The calculation of Vf, based on equation (4), yields Vf = 8.325 x $10^{-3}$ $m^{3}$/h, with the tower's dimensions (15 × 15× 40 cm), (Lx W x H) as in shown Fig. 5 was calculated to ensure uniform airstream dispersion and 0.4 m depth. Due to the better collecting efficiency of deeper-depth emission flows, particles have a longer residence time after passing through the packing.

This scrubber utilizes readily available materials, enabling small and large scale charcoal Makers to reduce pollutants to be emitted to the surroundings at a minimal cost. A sheet made up of metal having 15 cm × 15 cm × 1.30 m dimensions was employed to construct this pollutant control equipment. The lower part of the tower is covered with a hollow metallic coating that facilitates the upward passage of gas and the downward flow of liquid by confining gravel within the tower, where it is connected to the chimney at a location of 0.3 m from the tower's overall height. From a total of 1.30 m depth, a layer of gravel measuring 0.4 m in height was placed. Above this layer, there was a 60 cm depth of empty space, which was purposely left empty in order to manage the dispersion of water. The chimney serves to connect the kiln to the wet packed scrubber, facilitating the transportation of smoke. Additionally, an elevated water storing tank has been implemented above the tower to allow for the gravitational distribution of water during the showering process.

### 2.5 Wood preparation and carbonization

**Fig. 6:**
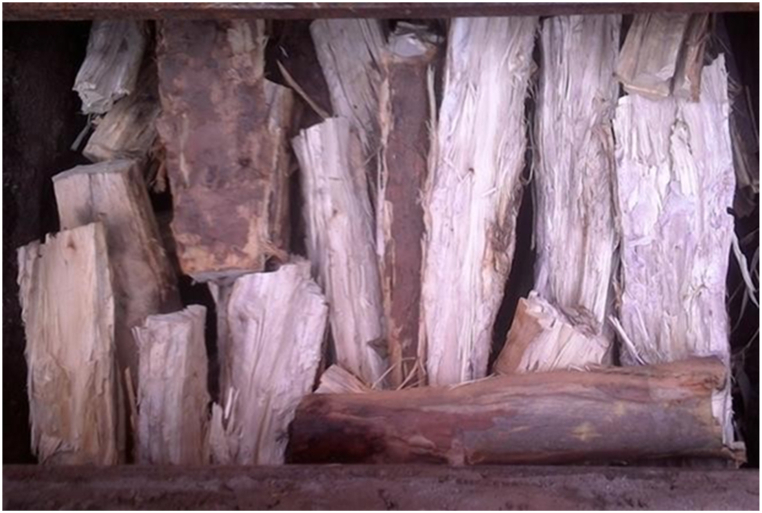
Wood sample feed into the kiln for the production of charcoal.

The eucalyptus globulus wood, which had been harvested at approximately 10 years of age, was subjected to cleaving in order to reduce its size. Subsequently, it was carefully stored under shelter to safeguard against the detrimental impact of moisture on the carbonization process, as illustrated in Fig. 6. For the purposes of this research, the wood samples exhibited an average moisture content of 29.5% and 223 kg weight. For the sake of creating a conducive environment for effective carbonization wood measuring 80–100 cm in length and 14–30 cm in diameter feed into charcoal production in orderly fashion.

To start the wood's initial ignition, wood was introduced into the kiln in an effective way. Due to its ease of usage and ability to preserve wood for retort, internal heating is the preferred method for this pilot project. Additionally, preference is given to internal heating over heating with recirculating gas. This preference stems from the fact that heating with recirculating gas requires a substantial investment and a workforce with specialized skills. The wood that has been size reduced is arranged in a vertical manner, while the remaining wood is placed horizontally on the uppermost layer of the vertical wood. The holes at the base are left unobstructed to allow for ignition and the inflow of air at the start of the carbonization process.

**Fig. 7:**
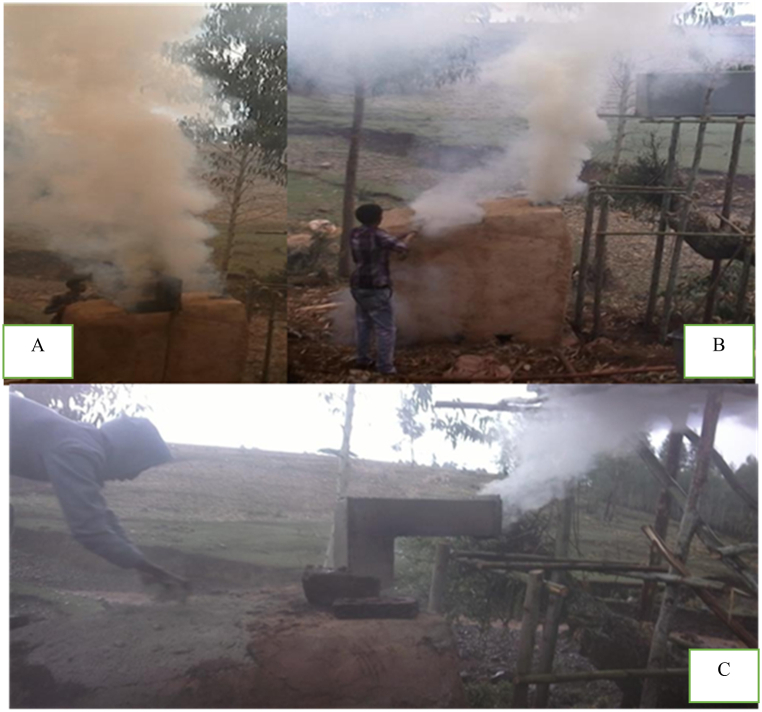
Initial stage of ignition (A), color of smoke after 15 min of wood carbonization (B) and final stage of carbonization (C) Color of smoke before treatment (A) and after treatment (B) Charcoal, CO, CO_2,_ CH_4_, PM, tars, ethane and heavy metals and H_2_O are the typical products and byproducts of charcoal producing activities.

In this procedure, wood was lit for 10–15 min, and the wood burned at base holes wherein the top aperture was closed. Then bricks are positioned onto the upper door and coated with a slurry consisting of a combination of mud and ash in order to safeguard against the escape of heat and volatile materials. The process of wood carbonization was permitted until the emission of bluish smoke from the chimney. Thereafter, the air inlets, which are holes, were obstructed by bricks and a combination of mud and ash. Subsequently, the process of carbonization was carried out for a duration of 16 h. This process commenced at 2:14 p.m. and was continued throughout the night, concluding the following day at 6:20 a.m. Then, the chimney was sealed shut and left to cool. Finally, the kiln's side door was opened, and the cooled charcoal was discharged. Soil was employed as a protective measure against the re-ignition of the charcoal upon exposure to air, as illustrated in Fig. 7 (A, B).

### 2.6 Emissions and control method

**Fig. 8:**
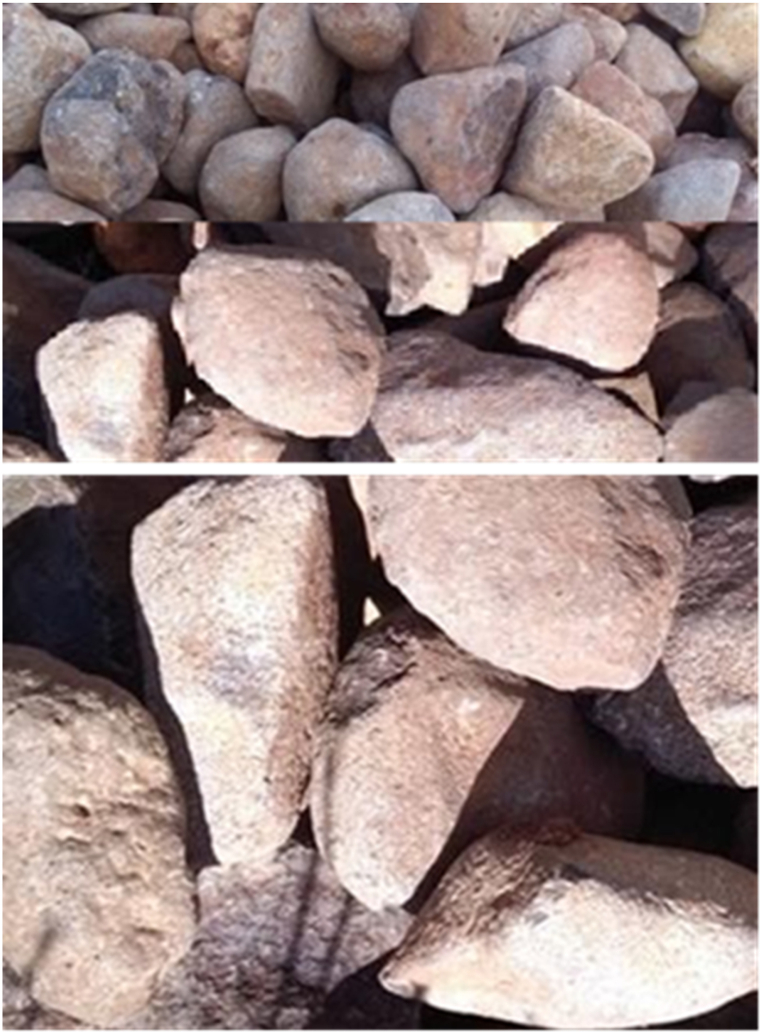
Gravel arranged from top to bottom for emitted pollutant control during carbonization.

A wet packed scrubber was specifically designed using gravel owing to its remarkable efficiency in collecting particulate matter and its exceptional resistance to corrosion. The gravels were strategically arranged in the scrubber with varying sizes and configurations. For instance, a gravel with an aggregate size of 48–60 mm was positioned at the bottom of the tower as shown in Fig. 8, thereby facilitating the flow of smoke through the gravel with a slight challenge. Furthermore, in the middle part of the tower, a gravel with an aggregate size of 27–33 mm was placed to decrease the gap between the gravels, ensuring optimal performance. Finally, a gravel with an aggregate size of 16–20 mm was positioned above the aforementioned layers.

Thus, three tests were carried out using the configuration mentioned above: In the first, 20 cm of thickness was fixed to the bottom (48–60 mm), 10 cm was fixed to the center (27–33 mm), and 10 cm was fixed to the top (16–20 mm). As a result, outgassing entered the tower from the stack. Thereafter, it comes into contact with the larger size gravel which partially obstructs the gas flow but makes passage difficult. The middle and upper sections of the gas flow continue, making the upward gas flow more difficult. In the second test, the larger size (48–60 mm) was completely removed from the bottom, and the two sizes (27–33 mm and 16–20 mm) were arranged with equal (20 cm) thickness between them. Removing the two types (27–33 mm and 16–20 mm) and filling the entire height (40 cm) with larger-sized (48–60 mm) gravels allowed for testing of the third. As a result, gases that have been emitted experience a reduction in their velocity, and the particulate matter becomes ensnared within the gravels. Simultaneously, water or a solution is directed downwards by a shower head, serving to cleanse each gravel and assimilate substances from the stream of gases.

## 3 Result and discussion

### 3.1 Kiln characteristics

The 1.25 m^3^ volumes and 18 cm wall thickness used in the construction of the brick kiln. This thickness does not meet the standard thickness required by kiln, which meant that in order to achieve proper carbonization; the kiln needed to be constructed using double bricks. For small-scale production, the majority of research reports utilized 30 cm–40 cm, and for commercial-scale production, 40 cm–50 cm. The efficiency of the kiln is impacted by thin wall heat loss. Due to considerable air escaping into the kiln, it consequently decreases charcoal yield and raises CO_2_ emissions. The Adam retort, with a 30–40% conversion rate, is one of the most effective kilns. Comparable to the Adam retort kiln, the currently constructed brick kiln has a conversion capacity of 38% at the specified condition. This might be as a result of the double-brick kiln wall's construction. This kiln still has a higher efficiency than conventional earth kilns (15–20%) [32], cassamance kilns (26–30%) [33], drum kilns (20–30%) [34], and portable metal kilns (26–30) [35]. This kiln is preferred by individuals who had been producing charcoal using the low-efficient kilns mentioned above and other similar ones due to its low cost.

### 3.2 Charcoal characteristics

It took 16 h to carbonise the material, then another day to cool it. A total of 2.6 bags weighing 31.6 kg each were used to collect the 85 kg of charcoal produced by this technique. Only core charcoal with an aggregate size of over 20 mm is taken into account for this weight. Ash weighted 1.8 kg, and the amount of charcoal having an aggregate size of less than 20 mm (fine charcoal) was 3.5 kg. As evidenced by here, eucalyptus globules make excellent firewood for making charcoal. This charcoal has a moisture content of 9%, which is within a range of typical charcoal moisture content reported to be (5–15%). Additionally, the ash content of currently produced charcoal was found to be 1.5% indicating a good charcoal. Basically, charcoal with lower ash content has higher heating value. Furthermore, since the charcoal yield from the Eucalyptus globules is deemed sufficient, it is possible to replace non-regenerating plants for the production of charcoal. The heating value of the charcoal produced from eucalyptus tree was determined to be 27.53 MJ/kg. This calorific value is comparable with the eucalyptus globules based charcoal's heating value found in the published literature. For instance, Jorge Gominho et al. reported 18.9 MJ/kg [36], Felix Charvet and coauthors reported 27.6 MJ/kg [37], Luciano E. Chiang et al.14.56 [38], heating value (26.69 MJ/kg) reported by Yoseph Shiferaw and coauthors [39].

### 3.3 Emission and wet packed scrubber characteristics

Charcoal production needs emission control technology, because there are certain harmful pollutants being emitted during the carbonization process. Accordingly, the wet packed scrubber was integrated to the charcoal producing kiln aiming to treat the pollutants generated such as particulate matter, gases, water vapor and heavy oil. Fundamentally, as pollutants like particulate matter, heavy oil and water vapor pass through the wet packed scrubber much of them are being captured and washed by water shower. On the other hand, gases are absorbed by water droplets. The amount of the pollutants emitted from charcoal producing kiln was measured before they are being treated using a wet scrubber in order to evaluate the removal capability of the wet scrubber as shown in Table 1. Before treatment, the concentrations of exhaust smoke in terms of CO, and CO_2_ were found to be 5.6 and 18.91% respectively.

**Table 1:** Quantity of pollutants emitted before being subjected to treatment.

HC	**CO**	${CO}_{2}$	**Time**
1959 ppm	5.6%	18.91%	03:02:21 p.m.

As the smoke changed to bluish colour, type and concentration of gases emitted during carbonization process was determined. This is due to the fact that, the dark/black smoke emitted during carbonization cannot provide accurate content of the pollutants for analysis. Additionally, following the ignition of the wood, that lasted approximately 15–30 min, the kiln released a dark or black smoke, which is not enough to determine the gas item and quantity accurately.

As shown in Table 2, gaseous pollutants like ${CO}_{2}$, CO and HC were detected before treatment. The concentration these pollutants are expressed in terms of ppm for hydrocarbons, whereas the percentages were used in order to quantify the status of pollutants being emitted. Normally, the gas detector being applied in this study can detect harmful pollutants like ${SO}_{X}$ and ${NO}_{X}$. However, ${SO}_{X}$ and ${NO}_{X}$ had not been detected in this study showing absence of these pollutants in the emitted smoke. This suggested that the eucalyptus globules derived charcoal is free of ${SO}_{X}$ and ${NO}_{X}$. However, it is common to find these pollutants in the solid waste derived charcoal because of the mixture of these chemicals bearing materials with solid wastes.

**Table 2:** Quantities of emitted pollutants after treatment.

Test	Gravel size	Thickness	HC(ppm)	CO (%)	CO_2_ (%)	Testing time
First	48–60 mm bottom 27–33 mm middle 16–20 mm top	20 cm 10 cm, 10 cm	59	0.31	2.12	03:30:12 p.m.
Second	27–33 mm bottom 16–20 mm top	20 cm 20 cm	43	0.04	0.28	03:40:51 p.m.
Third	48–60 mm full	40 cm	263	1.80	11.93	03:45:59 p.m.

**Fig. 9:**
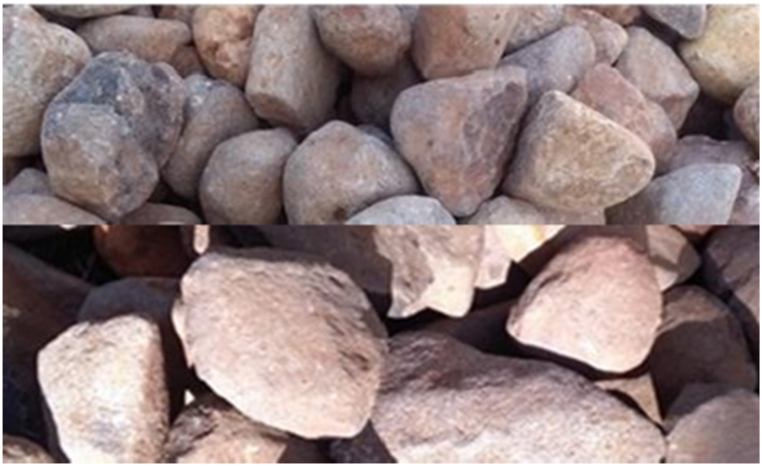
The two layers of wet scrubber applied for pollutant removal.

According to the initial test, CO has been cleared by 94.5% and has decreased to 1460.42 ppm, ${CO}_{2}$ has decreased to 58,246.6 ppm from 65,593 ppm, and HC has decreased to 1900 ppm from 1959 ppm. The results of the second test revealed that CO was 5.6% prior treatment and 0.04% after. This indicated that 1535 ppm of the total 1546 ppm of CO that was emitted was absorbed, or 99% of the CO. HC fell by 97.8%, resulting in 1916 ppm absorbed from 1959 ppm, while ${CO}_{2}$ declined by 98.5%, resulting in 64,622 ppm out of 65,593 ppm. In the third test, CO was lowered by 86.6% and 1696 from 1959 ppm, ${CO}_{2}$ was absorbed by 36.9% and 24,211 ppm, and CO was treated by 1049 ppm from 1546 ppm. According to the data above, the second test, in which gravels were arranged so that size 16 mm–20 mm gravels were attached at the top with a 20 cm thickness and size 27 mm–33 mm gravels were fixed at the bottom with a 20 cm thickness as shown in Fig. 9, has resulted in best results

By reducing their flow rate, the two layers facilitate the interaction of water and gases. Carbonic acid, a weak acid that dissociates into carbonic ions that are ready to react with Ca, Mg, CaO, and other elements and ions, is produced when water comes into contact with gases like CO and ${CO}_{2}$. Lime was utilized because it was cost effective to reduce the acidity of the effluent.

$$\text{CO}+H_{2}\;O↔{CO}_{2}+H_{2}\Delta H^{O}298=‐41.09\text{kJ}/\text{mol}$$

$${CO}_{2}\;\left(\text{aq}\right)+H_{2}\;O\;\left(l\right)\;↔\;H_{2}{CO}_{3}\left(\text{aq}\right)$$

The amount of ${CO}_{2}$ that is reduced by this method is 67.9 kg out of 69 kg. In contrast to cyclones that only filter PM, ineffective wet scrubbers had been employed for pollutant removal and fabric filters that only remove PM; this technique is more effective when layers of gravels are formed of smaller gravels. The fact that the packaging gravel can be recycled after a batch or several batches of manufacture is another benefit of this technology. The disadvantage of this technology is that stone, which is heavy and requires strong holding above, is used as packaging material

### 3.4 Cost analysis

The detailed cost analysis is depicted in Table 3 (equipment cot), and Table 4 (labour cost).

**Table 3:** Raw material cost.

Raw material	Unit	Amount	Unit price (ETB)	Total price (ETB)
Bricks	Pieces	500	5	2500
Metal sheet		3	260	720
Cement	kg	300	2.60	780
Sand	m^3^	3	260	780
Wood	m^3^	1.1	233	256
Total				5036

**Table 4:** Labour cost.

Duties	Number of person	Cost per person (ETB)	Total cost (ETB)
Kiln construction	2	150	600
Gravel collection	2	50	100
Wet packed scrubber construction	2	200	800
Total			1500

### 3.5 Comparative analysis

This affordable brick kiln with emission control technology offers several advantages over traditional methods of charcoal production. Firstly, the batch operation of the brick kiln ensures better control over the carbonization process, resulting in higher-quality charcoal with a moisture content of only 9% and an ash content of 1.5%. Traditional methods often yield charcoal with higher moisture and ash content, reducing its efficiency and heating value. Additionally, the emission control technology integrated into the brick kiln significantly reduces the release of harmful gases such as hydrocarbons, CO_2_, and CO by 97.8%, 98.5%, and 99%, respectively. In contrast, traditional methods often lack effective emission control measures, leading to increased air pollution and health hazards for workers.

The proposed brick kiln also offers advantages when compared to advanced methods of charcoal production such as a retort kiln. Retort kilns are known for their higher charcoal yield and better emission control compared to traditional methods [40]. However, this brick kiln with its emission control technology can achieve similar or even higher charcoal yields of 38%. Furthermore, the wet-packed scrubber in the brick kiln effectively removes harmful gases, making it comparable or even superior to retort kilns in terms of emission control. Additionally, the brick kiln is designed to be more affordable and accessible, allowing small-scale producers to adopt cleaner and more efficient charcoal production methods.

Pyrolysis is a more advanced method that involves the heat induced breakdown of biomass without the presence of oxygen. While pyrolysis offers advantages such as higher conversion efficiency and the production of valuable byproducts like bio-oil and syngas [41], it often requires complex and expensive equipment [42]. In contrast, this brick kiln provides a simpler and more cost-effective solution for charcoal production, particularly suitable for small-scale operations. The emission control technology integrated into the brick kiln ensures a significant reduction in harmful emissions, comparable to or even better than pyrolysis systems, making it an attractive alternative for sustainable charcoal production.

There are also other advanced methods for charcoal production, including electric kilns, retort kilns with gas circulation systems, and carbonization chambers with advanced control systems. These advanced methods often offer higher efficiency, precise control over the carbonization process, and reduced emissions. However, they also come with higher costs, technical complexity, and may require a larger scale of operation to be economically viable. This brick kiln with emission control technology bridges the gap between traditional methods and advanced systems by providing a cost-effective solution with effective emission control, making it a practical and feasible option for small-scale charcoal producers, such as communities in developing countries like Ethiopia.

### 3.6 Specific environmental benefits and drawbacks of the proposed kiln

The proposed brick kiln presents specific environmental benefits. Firstly, the integration of a wet-packed scrubber significantly reduces emissions of hydrocarbons, CO_2_, and CO by 97.8%, 98.5%, and 99%, respectively. This reduction in emissions contributes to improved air quality and mitigates the environmental impact associated with traditional charcoal production methods. The effective control of these gases helps minimize the contribution to climate change and reduces the potential health risks for workers and nearby communities.

Moreover, the use of Eucalyptus Globulus wood as biomass for charcoal production offers potential environmental benefits. Eucalyptus Globulus is a fast-growing species, making it a renewable and sustainable source of biomass. By utilizing this wood for charcoal production, the brick kiln reduces the demand for traditional timber resources, which often involves deforestation and depletion of natural forests. The sustainable use of Eucalyptus Globulus wood helps in preserving biodiversity, maintaining ecosystem services, and promoting sustainable land management practices.

However, it is important to consider some drawbacks of the proposed brick kiln. The production of charcoal, even with emission control measures, still results in the release of greenhouse gases, although at reduced levels. The process of carbonization also requires energy, typically derived from the combustion of biomass, which contributes to carbon emissions. Additionally, the use of Eucalyptus Globulus wood as the primary feedstock raises concerns about the potential impact on local ecosystems and biodiversity if large-scale plantations are established. Thus, careful monitoring and sustainable sourcing of the wood feedstock are essential to minimize any negative environmental impacts.

### 3.7 Long-term environmental impact and sustainability of the proposed brick kiln

The proposed brick kiln's long-term environmental impact and sustainability depend on several factors. Continuous usage of the affordable brick kiln with emission control technology can lead to sustained reductions in harmful emissions and improved air quality. The effective removal of hydrocarbons, CO_2_, and CO contributes to mitigating climate change as well as reducing the environmental and health impacts associated with conventional charcoal production. The sustainable use of Eucalyptus Globulus wood as biomass helps in maintaining the balance of ecosystems and preserving biodiversity.

To ensure long-term sustainability, it is crucial to address potential issues that may arise from continuous usage. Regular maintenance and monitoring of the brick kiln and its emission control systems are necessary to ensure their optimal performance and effectiveness. Proper training and education of charcoal producers on the operation and maintenance of the brick kiln will facilitate its long-term sustainability. Moreover, research and development efforts should focus on further improving the efficiency and environmental performance of the proposed brick kiln. This may involve optimizing the emission control technology and integrating renewable energy sources for the kiln's energy requirements. Continuous innovation and technological advancements will contribute to the long-term environmental sustainability of the proposed brick kiln.

## 4 Conclusion

In this study, a brick kiln was designed and fabricated with enhanced emission control technology for Eucalyptus globules-derived charcoal production. The charcoal produced was found to have characteristics of low moisture content and ash content having 38% charcoal yield. The characteristics of charcoal proved that the Eucalyptus globules wood-derived charcoal to be a good energy material. On the other hand, the gravel-based wet scrubber was integrated into the brick kiln and able to significantly decrease the pollutants concentrations to be emitted to the environment. The removal capability of the wet scrubber is determined to be 99%, 98.5% and 97.8% for CO, ${CO}_{2}$ and HC respectively. Additionally, this charcoal production brick kiln can be constructed at a modest initial cost of 7080 ETB which makes it feasible, simple to construct and adaptable. However, further researches such as temperature and air flow rate optimization and more detailed data on the types and quantities of gases emitted before and after treatment would enhance the understanding of the environmental impact**.**

## Data availability

All data generated during investigation is included in the manuscript.

## Funding

No funding was received for this work.

## CRediT authorship contribution statement

**Zelalem Getahun:** Validation, Methodology, Investigation, Conceptualization. **Mikiyas Abewaa:** Writing – review & editing, Writing – original draft, Formal analysis, Data curation. **Ashagrie Mengistu:** Writing – review & editing, Writing – original draft. **Eba Adino:** Writing – review & editing, Writing – original draft. **Kumera Kontu:** Writing – review & editing, Writing – original draft. **Kenatu Angassa:** Supervision. **Amare Tiruneh:** Supervision. **Jemal Abdu:** Supervision.

## Declaration of competing interest

The authors declare that they have no known competing financial interests or personal relationships that could have appeared to influence the work reported in this paper.
